# A Hygienic Necessity

**Published:** 1890-01

**Authors:** 


### A HYGIENIC NECESSITY.

It is interesting to a lover of hygienic reform to note the increasing interest of women in the subject of healthful dress. Though far too early in the day to expect radical reform in the garments of the many, yet the living example of the minorty is as a precious leaven which, soon or late may permeate the whole. The trend of the times in this regard is seen in the free discussion of women's, and even girls' clubs throughout the country; in the new toleration accorded to unpleasant facts and figures concerning it, and in the unconscious reaching out of women for more comfort in dress, or rather, more immunity from discomfort. This last, most significant sign of all, is general; at least among those who comprise the respectable middle class. It is the undoubted outcome of that other reaching up and out of women, into professional and business life; to compete with man as she must, when she enters his long indisputed domain, she must, like him, wear garments which do not fetter or impede. Perhaps her mental aspirations will prove her physical salvation, after all, for the more she plans to do and to be, the more does she see that she must have *health*, in order to compass it. Experience, that disagreeable but always successful schoolmaster, is slowly bringing her to see that she can do little, and *be* still less, with all that dreadful weight of skirts hanging from her hips. After carrying from fifteen to twenty pounds, the removal of ten or fifteen pounds is indeed a great reform, but it will shortly be forced upon her understanding that absolute hygiene does not permit *one added ounce* of weight upon the hips.

She begins to see that she must decrease the number of bands at the waist, and therefore the number of skirts must be diminished ; one of lightish material with warm, union under-garments, being sufficient for comfort. The dress, too, for the same reason, will have to be made of light weight goods, and in one piece ; the skirt fashioned in severe simplicity, to avoid extra weight. And lastly, the heavy winter cloak, with tight-fitting waist, and skirts to the bottom of the dress, will have to be modified or banished. In fact all this modern costume, with its scant provision for warmth, its thin, tight boots, and its immense drag upon the hips, will have to be relegated to the purely fashionable woman ; by and by, when it gets to be unfashionable for women to be idle and useless, even she will discard it.—E. L. S. in *Good Health.*

